# Characteristics of deceased subjects transported to a postmortem imaging center due to unusual death related to epilepsy

**DOI:** 10.1002/epi4.12891

**Published:** 2024-02-01

**Authors:** Yoshiki Ito, Nobuhiro Hata, Satoshi Maesawa, Takafumi Tanei, Tomotaka Ishizaki, Manabu Mutoh, Miki Hashida, Yutaka Kobayashi, Ryuta Saito

**Affiliations:** ^1^ Department of Neurosurgery Nagoya University School of Medicine Nagoya Aichi Japan; ^2^ Department of Neurosurgery, Sakura General Hospital Aichi Japan; ^3^ Department of Surgery, Sakura General Hospital Aichi Japan

**Keywords:** cerebrovascular disease, drowning, mortality risk, myocardial damage, SUDEP

## Abstract

**Objective:**

Patients with epilepsy have high risk of experiencing uncommon causes of death. This study aimed to evaluate patients who underwent unusual deaths related to epilepsy and identify factors that may contribute to these deaths and may also include sudden unexpected death in epilepsy (SUDEP).

**Methods:**

We analyzed 5291 cases in which a postmortem imaging (PMI) study was performed using plane CT, because of an unexplained death. A rapid troponin T assay was performed using peripheral blood samples. Clinical information including the cause of death suspected by the attending physician, body position, place of death, medical history, and antiseizure medications was evaluated.

**Results:**

A total of 132 (2.6%) patients had an obvious history of epilepsy, while 5159 individuals had no history of epilepsy (97.4%). Cerebrovascular disease was the cause of death in 1.6% of patients in the group with epilepsy, and this was significantly lower than that in the non‐epilepsy group. However, drowning was significantly higher (9.1% vs. 4.4%). Unspecified cause of death was significantly more frequent in the epilepsy group (78.0% vs. 57.8%). Furthermore, the proportion of patients who demonstrated elevation of troponin T levels without prior cardiac disease was significantly higher in the epilepsy group (37.9% vs. 31.1%). At discovery of death, prone position was dominant (30.3%), with deaths occurring most commonly in the bedroom (49.2%). No antiseizure medication had been prescribed in 12% of cases, while 29.5% of patients were taking multiple antiseizure medications.

**Significance:**

The prevalence of epilepsy in individuals experiencing unusual death was higher than in the general population. Despite PMI studies, no definitive cause of death was identified in a significant proportion of cases. The high troponin T levels may be explained by long intervals between death and examination or by higher incidence of myocardial damage at the time of death.

**Plain Language Summary:**

This study investigated unusual deaths in epilepsy patients, analyzing 5291 postmortem imaging cases. The results showed that 132 cases (2.6%) had a clear history of epilepsy. In these cases, only 22% cases were explained after postmortem examination, which is less than in non‐epilepsy group (42.2%). Cerebrovascular disease was less common in the epilepsy group, while drowning was more common. Elevated troponin T levels, which suggest possibility of myocardial damage or long intervals between death and examination, were also more frequent in the epilepsy group compared to non‐epilepsy group.

**Key points**

- Of 5291 of unusual death cases, 132 (2.6%) with obvious history of epilepsy were analyzed retrospectively.
- Postmortem examination confirmed the cause of death in 29 cases (22.0%) in the epilepsy group, fewer than in non‐epilepsy group (42.2%).
- Cerebrovascular diseases were significantly less frequent in the epilepsy group, whereas drowning was significantly more frequent.
- Unspecified cause of death in those with elevated troponin T levels without prior cardiac disease was more frequent in epilepsy group.

## 1 INTRODUCTION

Patients with epilepsy are at significant risk of experiencing uncommon causes of death.^1^ Specifically, sudden unexpected death in epilepsy (SUDEP) refers to the unexpected and non‐traumatic death of patients with epilepsy, wherein postmortem examination reveals no anatomical or toxicological cause of death.^2^, ^3^ SUDEP accounts for 18% of epilepsy‐related deaths and is the most common cause of death in patients with chronic refractory epilepsy.^2^, ^4^, ^5^ While a longer duration of epilepsy and use of multiple antiseizure medications have been identified as risk factors for SUDEP,^4^, ^5^, ^6^, ^7^ the most significant risk factor is said to be focal to bilateral or generalized tonic–clonic seizures.^7^, ^8^, ^9^, ^10^ It has been established that the prevention of SUDEP is dependent on seizure prevention.^11^, ^12^ Nevertheless, the direct cause of SUDEP remains unknown.

The Postmortem Imaging (PMI) Center in Sakura General Hospital, located in the northern region of Aichi Prefecture in Japan, generally undertakes postmortem examinations when the cause of death is unknown, involving plane CT scans of the head and trunk. This includes cases of sudden death brought to the hospital by emergency medical services, sudden deaths that occurred in the hospital, and unusual deaths that occurred in all areas of Aichi Prefecture and parts of the adjacent Gifu Prefecture, as requested by the police. “Unusual death” is a death of unknown cause in an individual that is found deceased and reported to the police. More than nine million people live in these regions, with 700–800 cases requiring PMI studies at our hospital annually. In addition to PMI, in cases lacking an obvious external cause or when other internal causes of death are evident (e.g., brain disease), a rapid troponin T assay is performed using peripheral blood samples as supplementary information. The utility of PMI, which is the examination of body images to determine the cause of unusual deaths, has been widely recognized in recent years.^13^

A proportion of the cases that were evaluated using PMI in our hospital had a history of epilepsy. This study aimed to review cases of unusual death related to epilepsy and to explore factors that may contribute to these deaths and may also include SUDEP. We extracted cases with a history of epilepsy from those who had undergone PMI in the past 7 years and examined their characteristics, including presumed cause of death, situation at the time of discovery, medication status, and life background.

## 2 METHODS

Between April 2014 and March 2021, a PMI study was performed on 5514 cases at Sakura General Hospital (Aichi, Japan) to determine the cause of death and prepare autopsy reports. Among these, 223 cases were not issued an autopsy report at the center for several reasons related to police investigations. In those cases, only CT images were acquired and provided to the police, while no clinical information could be obtained. Therefore, those cases were excluded from the study, and the remaining 5291 cases were included. This retrospective study was approved by the Ethics Committee of Sakura General Hospital. All subjects were deceased at the time of the study; therefore, opt‐out consent was obtained from their families. No case was excluded by the opt‐out declaration.

Upon receiving a request from the police, a body of a person who experienced unusual death was brought to the PMI center of Sakura General Hospital. Clinical information was obtained from the police official assigned to the case, including sex, age, medical history, and medication status (including antiseizure medications). The police interviewed family members, cohabitants, and those who found the body for information. A direct interview with them by the attending physician at the center was not allowed for reasons related to the police investigation. Information regarding the discovery of the body, including body position, place of death, and presence or absence of wounds on the body's surface, was also provided by the police. This study included only cases in which the person was found dead and reported to the police. No cases of sudden death witnessed directly were included. Therefore, the lapse between the moment of death and the discovery of the body was unknown, and the time elapsed from the moment of death to the postmortem examination could not be evaluated.

Plane CT scanning was performed from the head to the knee (Aquilion Prime SP®, Canon Medical Systems) in all cases. Routine imaging conditions were as follows: X‐ray tube voltage, 120 kV; the X‐ray dose, 400 mAs; multi‐slice scanning with 0.5‐mm scanning slice thickness, and 5‐mm slice thickness for reconstruction. The head CT images were generally checked and interpreted by board‐certified neurosurgeons with expertise in autopsy imaging. Any significant findings such as hemorrhages, infarctions, traumatic changes, hydrocephalus, malformations, or tumors were documented. Radiologists read the images in other locations, and any findings in the lung, heart, aorta, stomach, small intestine, liver, gallbladder, bile duct, pancreas, spleen, kidney, prostate, uterus, ovary, lymph nodes, spine, and bone were documented. If the cause of death was still unclear based on past medical history, the circumstances at the time of discovery, and the postmortem CT images, a peripheral blood sample was collected and tested with a rapid troponin T‐test kit (TROP T Sensitive®, Roche Diagnostics Japan, Japan). This rapid kit result was considered positive if the troponin T level was equivalent to a quantitative level of 0.1 ng/mL or higher, in contrast to 99 percentile value of troponin T in healthy subjects corresponding to 0.014 ng/mL. The kit does not provide quantitative information. Early testing after cardiac infarction using this kit may demonstrate false‐negative results, because troponin T level reaches the minimum detection sensitivity in the assay at 3–5 h after the onset of myocardial infarction. Although the elapsed time after death was unknown in each case in our study, most of them took some time to be discovered, and were brought in after on‐site investigation by police; therefore, the likelihood of false‐negative Troponin T results was low. Following an extended postmortem interval, a time‐correlated increase in troponin T has been observed in cases of myocardial infarction and asphyxia, while such elevation in troponin T is not evident in death due to other causes.^14^ The result of the troponin T‐test was only used as supplementary information, and not to determine the cause of the death. After a comprehensive evaluation of all findings, an autopsy report was created by the attending physician at the PMI center. When the cause of death could be determined, it was included in the report (listed in Table 1); when the cause was not obvious, the death was classified as being due to “unspecified cause,” which corresponds to R99 in the International Classification of Diseases, 10th Edition (ICD‐10).

**TABLE 1:** The characteristics of subjects who underwent PMI studies in Sakura General Hospital (2017–2021).

	Epilepsy group		Non‐epilepsy group		
	*n* = 132 (2.6%)		*n* = 5159 (97.4%)		
Variables					*p* value
Male sex	82	62.1%	3555	68.9%	0.090
Female sex	50	37.9%	1604	31.1%	
Age, years (mean ± SD)	44.9 ± 15.3		61.4 ± 18.1		<0.001

Cause of death	No.		No.		
Unspecified	103	78.0%	2983	57.8%	<0.001
Unspecified with high troponin T	70	53.0%	2113	41.0%	0.005
Asphyxiation	5	3.8%	91	1.8%	0.085
Drowning	12	9.1%	228	4.4%	0.011
Subarachnoid hemorrhage	1	0.8%	233	4.5%	0.038
Cerebral hemorrhage	1	0.8%	297	5.8%	0.013
Aortic dissection	1	0.8%	39	0.8%	0.998
Gastrointestinal bleeding	1	0.8%	41	0.8%	0.962
Peritonitis	1	0.8%	8	0.2%	0.097
Pneumonia	2	1.5%	108	2.1%	0.646
Adrenal insufficiency	1	0.8%	0	0.0%	
Drug overdose	1	0.8%	44	0.9%	0.906
Carbon monoxide poisoning	1	0.8%	28	0.5%	0.741
Head trauma	2	1.5%	47	0.9%	0.474
Cardiac tamponade	0	0.0%	152	2.9%	
Others	0	0.0%	860	16.7%	

Abbreviation: PMI, postmortem imaging.

The deceased patients who underwent PMI studies were categorized into two groups based on the presence or absence of an obvious history of epilepsy. We compared age, sex, and suspected cause of death between these two groups. The distribution of causes of death among 132 patients in the epilepsy group was compared with that among patients in the non‐epilepsy group. Furthermore, we analyzed the characteristics of the 132 patients in the epilepsy group in detail, including the body condition when their death was discovered and their antiepileptic medication status.

Statistical analyses were performed using IBM SPSS Statistics 27 and 29. Descriptive statistical methods and the chi‐square independence test were used to evaluate the data. Statistical significance was set at *p* < 0.05. For the continuous variable “age”, a *t*‐test was used. Statistical significance was set at *p* < 0.01.

## 3 RESULTS

Among subjects who underwent PMI studies, a total of 132 cases (2.6%) had an obvious history of epilepsy, and 5159 had no history of epilepsy (97.4%). Table 1 illustrates the results on sex, age, and cause of death in both the epilepsy and non‐epilepsy groups. The analysis demonstrated no significant difference in sex between the two groups (*p* = 0.09). However, the age at death was significantly lower in the epilepsy group (mean ± SD, 44.9 ± 15.3 years) than in the non‐epilepsy group (61.4 ± 18.1 years; *p* < 0.001).

**FIGURE 1:**
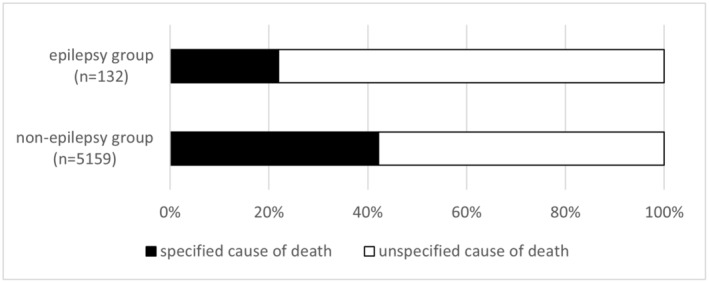
The proportion of cases within the epilepsy and non‐epilepsy groups where postmortem examination specified the cause of death.

Figure 1 shows the proportion of cases in both the epilepsy and non‐epilepsy groups in which the cause of death was specified by postmortem examination. Only 29 out of 132 cases (22.0%) in the epilepsy group had a specified cause of death, in contrast to 2176 out of 5159 (42.2%) in the non‐epilepsy group (odds ratio = 0.39; 95% confidence interval = 0.25–0.58). In the epilepsy group, postmortem CT contributed to the determination of the cause of death in 10 cases (7.6%). Causes included subarachnoid and cerebral hemorrhage, cardiac tamponade due to aortic dissection, peritonitis, gastrointestinal bleeding, and carbon monoxide poisoning. Head injury was reported in two cases, comprising acute subdural hematoma and cerebral contusion, and pneumonia was the cause of death in two cases. No deaths were attributed to cerebral infarction. In contrast, mortality due to hemorrhagic and obstructive cerebrovascular diseases was significantly higher in the non‐epilepsy group, accounting for 10.6%. Drowning was diagnosed based on a chest CT showing infiltration of water in the lungs and the situation at the time of discovery. The rate of drowning was significantly higher in the epilepsy group, with 12/132 (9.1%) cases reported (*p* = 0.011). Eight patients in the epilepsy group had their faces submerged in a bathtub. Asphyxiation was the cause of death in five cases (3.8%). In two cases, asphyxiation secondary to vomiting was suspected. One of these patients had been eating just before the sudden death, and the other vomited. In one case, the patient was found on the floor in a prone position. This information was collected from bystander or finder comments.

Patients in which no apparent cause of death could be determined were classified in the “unspecified cause of death” category. The epilepsy group comprised 103 (78.0%) cases with unspecified cause of death, which was significantly higher than the proportion in the non‐epilepsy group (57.8%; *p* < 0.001). Of those, five cases were initially diagnosed with “epilepsy” as the cause of death. In all five cases, the position and presence of a subcutaneous hematoma suggested that a seizure just before death caused the patient to fall. Although the fall was evident from the circumstances, this trauma alone was not considered to sufficient to have caused the death. We classified these cases as “unspecified cause of death” because other unknown factors may have contributed to the death. Elevation of troponin T without prior cardiac disease was observed in 70 cases, which was significantly higher (*p* = 0.005) than in the non‐epilepsy group.

Table 2 presents the locations and body position at the time of death in the epilepsy group. The bedroom was the most common site of death, accounting for 65 cases (49.2%). Meanwhile, 18 patients died in the bathroom, 7 with their faces submerged in water. Prone was the most common position at the time of death, comprising 40 cases (30.3%). Table 3 shows the medical background of the epileptic cohort, including 94 individuals (71.2%) with a history of diseases other than epilepsy. Organic brain diseases such as cerebrovascular disease, cerebral contusions, and brain tumors, were reported in 28 cases (21.2%); psychiatric disorders such as depression and schizophrenia, in 30 (22.7%); and mental retardation in 16 (12.1%). Chronic illnesses such as hypertension, diabetes, and asthma were observed in 37 cases, and some presented with more than one disease. Table 4 shows the type of antiepileptic medications prescribed and the number of concurrent medications administered to the epilepsy group. Sodium valproate had been prescribed in 43 cases, levetiracetam in 25, carbamazepine in 19, and lamotrigine in 11. In 16 cases, no antiseizure medications had been prescribed. Thirty‐nine cases (29.5%) were taking multiple antiseizure medications; and among them, 14 (10.6%) were taking three or more different medications.

**TABLE 2:** Place and position of death in subjects with epilepsy.

Place of death	No.
Toilet	4
Under the stairs hallway	2
Living room	11
Entrance	1
Office	1
Own room	15
Inside the house	6
Dining room	1
Bedroom	64
Detention room	1
Waterway	1
Kitchen	3
Bathroom	3
Bathtub	15
Corridor	3
Pachinko parlor	1

Position of death	No.
Right lateral decubitus position	9
Supine position	38
Supine position changing to lateral decubitus position	1
Left lateral decubitus position	4
Left lateral position	1
Sitting	6
Seiza position	1
Lateral decubitus position	2
Seated cross‐legged position	2
Long sitting position	1
Prone position	40
Unknown	27

**TABLE 3:** Medical history of subjects with epilepsy.

Medical history	No.
Brain diseases (Brain hemorrhage, infarction, tumor, trauma, etc.)	28
Psychiatric disorders	30
Pediatric diseases	16
Internal diseases (hypertension, diabetes, asthma, etc.)	37
Malignant diseases	4
Epilepsy only	38

**TABLE 4:** Antiseizure medication status in subjects with epilepsy.

Type of antiseizure medication	No.
Valproic acid	43
Carbamazepine	19
Phenytoin	7
Clonazepam	9
Phenobarbital	10
Zonisamide	5
Clobazam	3
Gabapentin	2
Topiramate	1
Levetiracetam	25
Lamotrigine	11
Lacosamide	3
Perampanel	4
Nitrazepam	4
Diazepam	1
None	16
Unknown	26

Number of antiseizure medications	No.
None	16
One drug	51
Two drugs	25
Three drugs	10
Four drugs	3
Five drugs	1
Unknown	26

## 4 DISCUSSION

### 4.1 Characteristics of unusual death in patients with epilepsy: Age and gender

**FIGURE 2:**
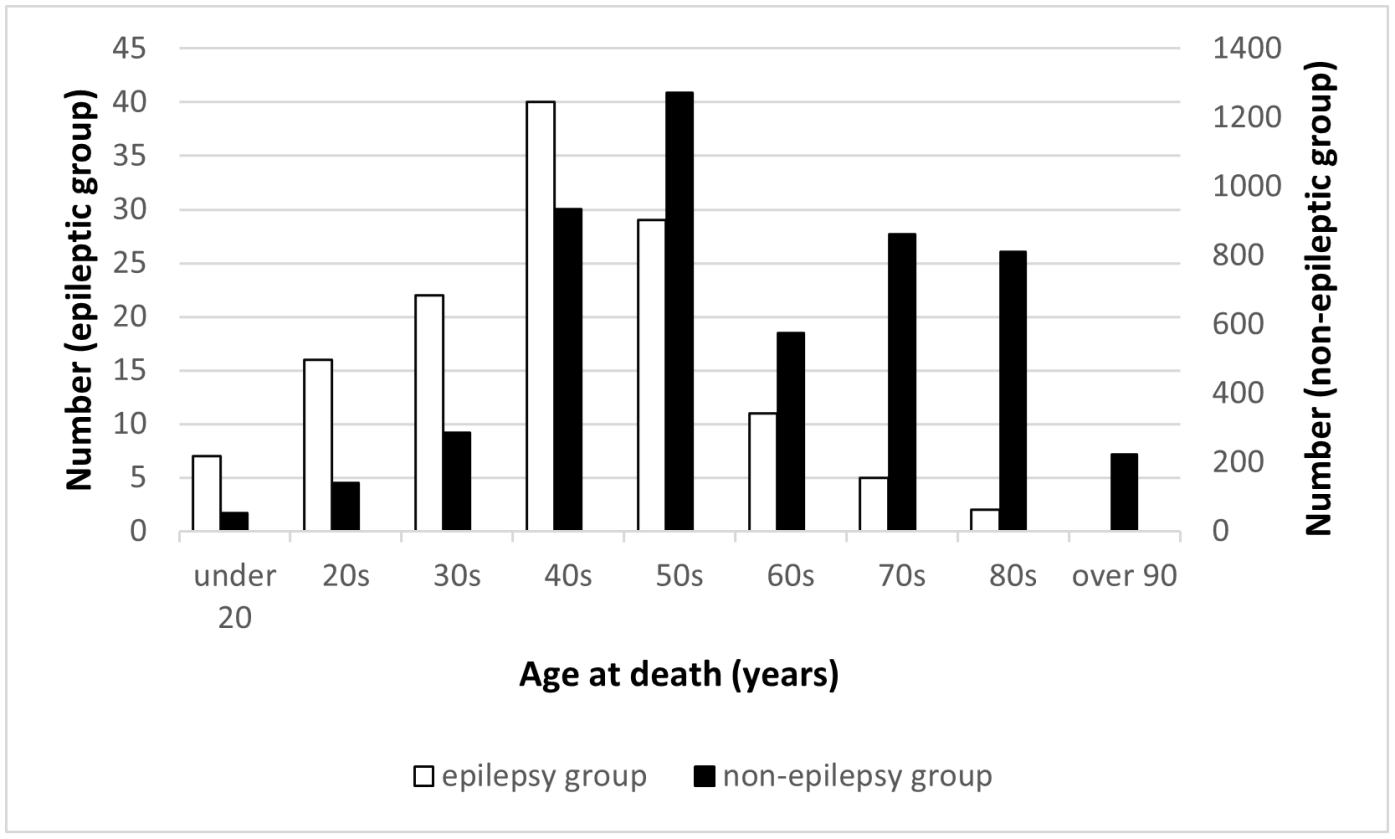
Age distribution in the postmortem imaging cases within epilepsy and non‐epilepsy groups. The non‐epilepsy group shows a bimodal distribution, which is similar to that in Japanese postmortem investigations, while the epilepsy group shows a unimodal distribution.

In this study, the prevalence of epilepsy among PMI cases was 2.49%, exceeding that in the general population (0.5–0.7%).^15^, ^16^ Although the prevalence of epilepsy is higher in men compared to women,^15^ we observed similar rates in both sexes. Furthermore, male sex has been identified as a risk factor for SUDEP.^7^, ^8^ The mean age at death in the epilepsy group was 44.9 ± 15.3 years, with the age distribution peaking in the 40s, indicating a younger age at death compared to the general population. The life expectancy of patients with epilepsy generally is 11–13 years shorter than in individuals without epilepsy.^17^ The highest prevalence of epilepsy is observed in the 25–49 years age group, declining with age.^16^ SUDEP incidence is low in early childhood, high in adolescence, peaks in young adulthood, and subsequently declines substantially.^9^, ^18^ However, the age distribution of postmortem examinations in Japan is bimodal, with the highest number of men in their 40s and 60s, and the number of women increasing with age, although regional differences exist.^19^, ^20^ In this study, the age distribution of PMI cases within the non‐epilepsy group was bimodal, similar to the results of Japanese postmortem investigations (Figure 2). When the age distribution among PMI cases within the epilepsy group was evaluated, a unimodal distribution with a peak in the 40s was observed (Figure 2). The age‐specific distribution of deaths in patients with epilepsy, as reported in other countries, generally peaks between 26 and 45 years and then declines.^21^ The age distribution in these patients is likely to be reflected in the number of abnormal deaths associated with epilepsy.

### 4.2 Characteristics of unusual death in patients with epilepsy: Cerebrovascular disease, drowning, asphyxia, and unspecified cause of death

In our cohort, cerebrovascular disease was a frequently identified cause of death in the non‐epilepsy group (10.6%). Recent statistics from the Tokyo Metropolitan Government regarding unusual deaths showed that cerebrovascular disease accounted for 9.8% of deaths overall,^22^ which was consistent with our findings. Regarding the epilepsy group in our study, we identified only one case of subarachnoid hemorrhage and one of cerebral hemorrhage as causes of death, which was significantly lower than in the non‐epilepsy group. The epilepsy group tended to be younger (in their 40s) and had fewer age‐related arteriosclerotic changes, which may have contributed to the lower incidence of cerebrovascular disease.

Drowning was significantly more frequent in the epilepsy group than in the non‐epilepsy group. Bain et al. reported that patients with epilepsy had a 10‐fold higher mortality rate from drowning than healthy individuals.^23^ In our study, the rate was 2.05 times higher. Our cohort was situated in an area with many rivers facing the sea, where outdoor water‐related deaths are relatively common, especially among those involved in the fishing industry or river‐based leisure activities. This may explain the significantly higher incidence of drowning in patients without epilepsy. Despite this, there were significantly more deaths by drowning in the epilepsy group. Of the 12 cases of drowning in the epilepsy group, 11 occurred in buildings. It has also been previously reported that most drowning events in patients with epilepsy occur in bathtubs,^23^ particularly in Japan where people commonly bathe in bathtubs.^24^ Despite the importance of antiepileptic drug management in drowning prevention, there is still a risk of death even when seizures are under control.^23^, ^25^ Patients with epilepsy should be fully informed about the risks of bathing, as drowning can take place at home if epileptic seizures occur during bathing.

Asphyxia was a common cause of death, but rates did not differ significantly between two groups. The most common causes of asphyxia in patients with epilepsy are airway obstruction due to vomiting and breathing difficulties due to uncontrolled posture during seizures. Kloster et al. reported that cases with SUDEP are predominantly found in the prone position, which may be a contributing factor to SUDEP.^26^ More than half of SUDEP cases occur during sleep,^27^ and the presence of seizures at night is associated with a 2.6‐fold increase in the risk of death.^28^

### 4.3 Characteristics of unusual death in patients with epilepsy: Unspecified cause of death and elevation of troponin T levels

In the group of patients with epilepsy, a considerable number of cases (78.0%) had no cause of death determined by postmortem imaging. The cause was, therefore, categorized as “unspecified cause of death,” which has been considered the equivalent of SUDEP. Deaths due to unspecified cause were considerably lower in the non‐epilepsy group (57.8%). The literature suggests that patients with epilepsy are 2.77 times more likely to experience an unnatural death than individuals with other diseases.^1^ Ficker et al. reported that the rate of unexpected sudden death from an unknown cause was approximately 24 times higher in patients with epilepsy than in healthy controls.^29^

Moreover, the cases with unspecified internal causes of death but positive peripheral blood troponin T were significantly more common in the epilepsy group. To explain this finding, several factors should be discussed. First of all, the sequence of tonic–clonic seizures ‐ central apnea ‐ asystole is the key pathophysiological cascade involved in SUDEP.^30^ However, it is unlikely to explain elevated troponin T levels, since the asystole associated with apnea does not directly induce myocardial damage.

Tonic–clonic seizures may directly damage the myocardium in the Takotsubo syndrome (neurogenic stunned myocardium). However, this syndrome is understudied and there is no proof so far that it contributes to SUDEP.^30^, ^31^, ^32^, ^33^ An alternative explanation concerns ischemia or prolonged arrhythmias (ventricular fibrillation). These conditions may also occur without seizures, for example, in the context of cardiovascular comorbidities^34^ or may be facilitated by myocardial damage in those with chronic refractory epilepsy (the “epileptic heart” hypothesis^35^, ^36^, ^37^).

In addition, several studies have established a connection between sympathetic activation and seizures.^38^ Nass et al.^39^ reported that high‐sensitivity troponin T was increased in the hours following an epileptic seizure and elevated in 26% of all patients. Blood dopamine levels are associated with troponin T levels, indicating that myocardial injury resulting from epileptic seizures is associated with strong sympathetic activation.

The patients with epilepsy have been found to have a higher cardiovascular risk and prevalence of cardiovascular disease compared to the general population^34^, ^40^, ^41^ as well as a higher risk of recurrent fatal arrhythmias and cardiovascular death.^42^, ^43^, ^44^ Nevertheless, others reported a 0.8‐fold reduction in the risk of death from cardiovascular disease in the epilepsy group.^45^ It is yet to be determined whether epileptic seizures trigger cardiovascular mortality.

This study included only cases in which the person was found dead and reported to the police. No cases of sudden death witnessed directly were included. Therefore, the lapse between the time of death and postmortem examination was not evaluated, and the high troponin T levels may be the result of a long laps. However, we observed significant differences between these two groups, and there is no reason to believe that this lapse was different between them. The higher rate of myocardial damage in epileptic patients is a more likely explanation.

### 4.4 Characteristics of unusual death in patients with epilepsy: Antiseizure medications

In terms of antiseizure medications, 10.6% of cases in our cohort used a combination of three or more drugs. Drug‐resistant epilepsy is characterized by seizures that cannot be controlled by three or more antiseizure medications, and is estimated to affect approximately 30% of all patients with epilepsy.^46^ While some studies suggest that the use of multiple antiseizure medications may be a risk factor for SUDEP,^4^, ^5^, ^6^, ^7^ others argue that it is not.^47^ Resistance to seizure medications may not have been a direct factor in our cohort. Furthermore, 12.1% of patients in our cohort had discontinued their antiepileptic medications. Psychiatric and intellectual disabilities may also affect medication adherence. Therefore, medication status, including non‐adherence to prescribed medications, should be considered.

### 4.5 Limitations

There are several limitations to this study. First, it did not encompass all cases, only those transported to our hospital by the police, which results in regional and age biases. Moreover, not all unusual deaths within the jurisdiction were included and the decision to perform postmortem imaging relied on the police. Second, details on the body condition, medical history, and medications were retrieved from the police, not from medical institutions. Third, multiple physicians were involved in determining the cause of death. The criteria for determining the cause of death depend on the physician handling the case. Therefore, if multiple physicians examined the same body, they may have determined different causes of death. Furthermore, the current study identified “unspecified internal disease” as a potential SUDEP. It is possible that some of these cases should have been diagnosed with a different cause of death. Fourth, it is unclear whether troponin T can serve as a biomarker of the cause of death. Rahimi et al. reported that there were no significant differences in troponin T levels between cases with different causes of death, and that it was not specific or useful as a postmortem cardiac biomarker.^48^ Therefore, it may be challenging to conclude that cardiac death is the most common cause of unusual death in patients with epilepsy. Troponin T levels are influenced by the time elapsed after myocardial injury, but information on the lapse between death and arrival of the body to the hospital was not available to us; therefore, the influence of this factor could not be assessed. Finally, this study was performed before the coronavirus pandemic, and data may not be representative of the current post pandemic situation.

## 5 CONCLUSIONS

Using data from a PMI center, we conducted a retrospective review and analysis of the factors influencing unusual death in patients with epilepsy. Among the cases of unusual death examined via PMI, patients with epilepsy were overrepresented. Despite undergoing PMI studies, no definitive cause of death was determined in a significant proportion of cases, and therefore, we suggest that the utility of postmortem CT is relatively limited in these patients. Cerebrovascular diseases were significantly less frequent as the cause of death in the epilepsy group, whereas drowning was significantly more frequent. The proportion of cases classified as unspecified cause of death was significantly higher in the epilepsy group than in the non‐epilepsy group. Considering the high rate of troponin T elevation in the epilepsy group, the underlying mechanism of SUDEP may be attributed to myocardial damage.

## AUTHOR CONTRIBUTIONS

Substantial contributions to the conception and design of the work: YI, NH, SM, YK, and RS. Acquisition and analysis of the data: YI, NH, and SM. Interpretation of data: YI, NH, SM, TT, TI, MM, MH, YK, and RS. Drafting the work and revising it critically for important intellectual content: YI, NH, and SM. All authors approved the final version of the article.

## CONFLICT OF INTEREST STATEMENT

None of the authors has any conflict of interest to disclose.

## ETHICS STATEMENT

We confirm that we have read the Journal's position on issues involved in ethical publication and affirm that this report is consistent with those guidelines. The studies involving human participants were reviewed and approved by the Local Ethics Committee of Sakura General Hospital. Opt‐out consent was obtained from the families of the deceased patients in this study.

## Data Availability

The original contributions presented in the study are included in the article / Supplementary material, further inquiries can be directed to the corresponding author.
